# A higher probability of subsequent stroke and ischemic heart disease in migraine patients: a longitudinal follow-up study in Korea

**DOI:** 10.1186/s10194-023-01632-y

**Published:** 2023-07-31

**Authors:** Mi Jung Kwon, Hyo Geun Choi, Yoo Hwan Kim, Joo-Hee Kim, Hyun Taek Rim, Heui Seung Lee, Jae Keun Oh, In Bok Chang, Joon Ho Song, Ji Hee Kim

**Affiliations:** 1 Department of Pathology, Hallym University College of Medicine, Anyang, Korea; 2 Suseoseoul ENT clinic, Seoul, Korea; 3 MD analytics, Seoul, Korea; 4 Department of Neurology, Hallym University College of Medicine, Anyang, Korea; 5 Division of Pulmonary, Allergy, and Critical Care Medicine, Department of Medicine, Hallym University College of Medicine, Anyang, Korea; 6 Department of Neurosurgery, Hallym University College of Medicine, Anyang, Korea

**Keywords:** Cardiovascular disease, Cerebrovascular disease, Heart failure, Ischemic heart disease, Migraine, Stroke

## Abstract

**Background:**

Whether migraine is related to the risk of cardiovascular diseases (CVDs) remains unclear. Therefore, we conducted a longitudinal follow-up study to address the association between migraine and the development of CVDs in Korea.

**Methods:**

Using data from the national health screening cohort, we included 45,246 patients diagnosed with migraine between 2002 and 2019 and age-, sex-, income-, and residential region-matched nonmigraine participants at a ratio of 1:4. Participants with previous CVDs were excluded. Cox proportional hazards regression models were used to estimate the hazard ratios of three CVDs, stroke, ischemic heart disease, and heart failure, in patients with migraine after adjusting for potential cardiovascular risk factors.

**Results:**

The incidence rate differences of stroke, ischemic heart disease, and heart failure among patients with migraine were 2.61, 1.69, and 0.11, respectively. The probability of developing stroke and ischemic heart disease in patients with migraine was significantly higher than that in controls after controlling for multiple confounders (adjusted hazard ratio [HR] = 1.35, 95% confidence interval [CI] = 1.31–1.39 and adjusted HR = 1.31, 95% CI = 1.26–1.35, respectively). However, when compared with the patients without migraine, patients with migraine did not have an increased HR of developing heart failure (adjusted HR = 1.01, 95% CI = 0.95–1.08). The overall migraine group, as well as groups stratified by migraine subtypes with and without aura, each showed a significantly higher probability of subsequent stroke and ischemic heart disease than the control group.

**Conclusions:**

Our longitudinal follow-up study demonstrated a significant association between the presence of migraine and the development of stroke and ischemic heart disease in Korea, even after adjusting for cardiovascular risk factors.

**Supplementary Information:**

The online version contains supplementary material available at 10.1186/s10194-023-01632-y.

## Background

Migraine, a chronic disorder with episodic headache attacks, is a frequent and debilitating neurological disorder that has an estimated 1-year prevalence of 15% worldwide [1]. Migraine manifests as recurrent attacks that last 4–72 h and have certain accompanying symptoms, such as nausea, phonophobia, and photophobia [2]. In addition, this headache is commonly throbbing, unilateral, pulsating, and moderate to severe in intensity and can be aggravated by ordinary physical activities. There are two main types of migraine: migraine with aura and migraine without aura, the latter subtype being the more common of the two [3]. Approximately 1 in 3 patients with migraine experience aura, which can be presented by visual, auditory, somatosensory, motor, language, or brainstem disturbances [4]. The wide range of symptoms and neurological disturbances observed during migraine attack suggests the involvement of multiple neural networks [5]. Notwithstanding its high prevalence and considerable disability rate, the pathophysiology of migraine has yet to be completely clarified.

Cardiovascular disease (CVD) is the major cause of death and a chief contributor to disability globally. CVD is a general term for a group of disorders affecting the heart or blood vessels and is usually includes coronary heart disease, cerebrovascular disease, peripheral arterial disease, rheumatic and congenital heart diseases and venous thromboembolism [6]. Although CVD may directly arise from different etiologies, risk factors related to the development of atherosclerosis are crucial because atherosclerosis is a common denominator in the pathophysiology of CVD. It involves many factors, including dyslipidemia, immunologic phenomena, inflammation, and endothelial dysfunction [7].

Growing awareness of the relationship between migraine and blood vessels, i.e., the vascular properties of migraine, such as abnormal reactivity of meningeal blood vessels [8, 9], has led to speculation that migraine and CVD could share pathological pathways. Furthermore, reports of cortical spreading depression, hypercoagulation, endothelial dysfunction, shared genetic risk, vasospasm, or a higher prevalence of cardiovascular risk factors among patients with migraine could support a close association between migraine and CVD events [10–12].

Based on shared potential mechanisms that were explained in the pathogenesis of migraine and CVD, population-based studies have been broadly conducted to determine whether migraine, particularly migraine with aura, is related to an increased risk of CVD [13, 14]. However, others have shown no significant associations [15], and most data have been examined more frequently among women [16, 17].

In light of this background, we sought to identify the association between migraine and the development of CVD after controlling for various cardiovascular risk factors in this longitudinal cohort study through a nationwide health screening database in Korea.

## Methods

### Data source

We conducted a retrospective cohort study using Korean National Health Insurance Service (NHIS)-Health Screening Cohort data, which is a cohort of subjects over age 40 who participated in bi-annual complimentary health screening programs provided by the NHIS in Korea. The characteristics of the Korean National Health Insurance Service (NHIS)-Health Screening Cohort data used in this study are described in detail in other research [18].

This study was approved by the ethics committee of Hallym University (2019-10-023). A waiver of written informed consent was approved by the Institutional Review Board. All procedures and analyses in this study complied with the guidelines and regulations of the ethics committee of Hallym University.

### Study design and participants

This study was conducted to investigate the association of migraine with the subsequent development of CVDs using data from the NHIS-Health Screening Cohort. The initial migraine group consisted of individuals who were diagnosed with migraine without aura (International Classification of Disease, Tenth Revision [ICD-10] code G430) and migraine with aura (ICD-10 code G431) at a minimum of 2 clinic visits between 2002 and 2019 (*n* = 54,877). Participants who were given a diagnosis of migraine (ICD-10 code G430 or G431) in 2002 (*n* = 6,479) were excluded from the analysis so that only newly diagnosed migraine cases were included. Participants who had no records of body mass index (BMI, *n* = 4), fasting blood glucose (*n* = 3), or total cholesterol level (*n* = 2) were also excluded. Additionally, those who had a history of CVD before the index date were excluded from the group (*n* = 3137). From 514,866 participants with 895,300,177 medical claim codes in the database, 45,246 patients remained in the migraine group for the matching process with the control group.

The initial control pool consisted of individuals who were not included in the initial migraine group during the period between 2002 and 2019 (*n* = 459,989). Patients who had been diagnosed with migraine once were excluded from the control group (*n* = 50,379). Therefore, a total of 180,984 individuals were included in the control group.

We matched migraine cases to controls based on age, sex, income and residential region. Four controls were selected for every migraine case. The included control participants were sorted using a random number order and then selected from top to bottom in turn. The index date was defined as the date of the first clinic visit that led to a diagnosis of migraine. Control participants were given the same index date as their respective matched patient in the migraine group (Additional file 1).

**Fig. 1:**
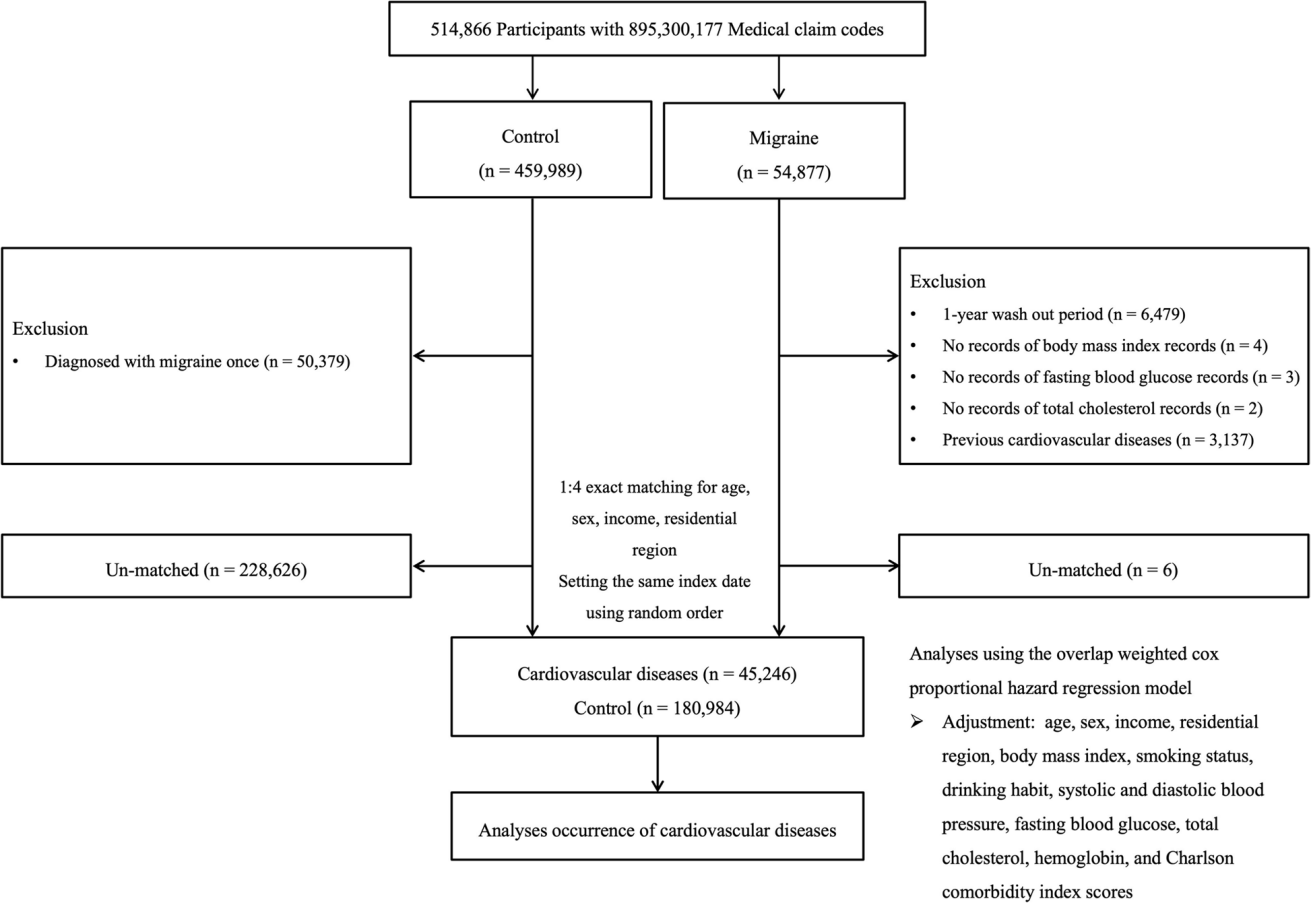
Flowchart illustrating the enrollment process of the migraine group and matched control group. Among 514,866 participants, 45,246 eligible patients with migraine were matched with 180,984 controls

The primary outcome was incident CVD during the follow-up period as defined by the following ICD-10 codes: stroke (I60-I69), ischemic heart disease (I20-I25), and heart failure (ICD code I50). We only included participants who were admitted two or more days with the appropriate claims or who died due to CVD, as described in our earlier study [19]. Additionally, participants who were given a diagnosis of CVD prior to the index date in both groups and who died prior to the index date in the control group were excluded from the study. Accordingly, 3,137 participants with prior CVD were excluded from the migraine group. Finally, 45,246 migraine participants and 180,984 matched control participants remained in the final migraine group and the final control group for the analyses (Fig. 1).

### Covariates

Details of participants’ age, sex, income status, residential region, BMI, smoking status, drinking habits, blood pressure, fasting blood glucose, total cholesterol, hemoglobin, and Charlson Comorbidity Index (CCI) were obtained from the NHIS database (Additional file 2). We considered these covariates as confounding factors for the association between migraine and CVD. Participants were classified into 10 age groups at 5-year intervals, and 5 income groups were divided from class 1 (lowest income) to class 5 (highest income). The residential region was divided into urban and rural areas according to our previous study [20]. Obesity as determined by BMI (kg/m^2^), smoking status, and drinking habits were also categorized in the same way as in our previous study [21]. Information on participants’ systolic blood pressure (SBP, mmHg), diastolic blood pressure (DBP, mmHg), fasting blood glucose (mg/dL), total cholesterol (mg/dL), and hemoglobin (HMG, g/dL) was extracted. CCI is assessed based on 17 comorbidities to measure disease burden. A score was given to each participant depending on the severity and number of diseases, and it was analyzed as a continuous variable (0 [no comorbidities] to 29 [multiple comorbidities]) [22]. Cerebrovascular disease, congestive heart failure, and acute myocardial infarction were excluded from the CCI score calculation for this study.

### Statistical analyses

We used propensity score (PS) overlap weighting to ensure that covariates in the two groups were balanced and that the sample size was effective. This involved calculating the PS for each participant using multivariable logistic regression and then using that score to weight participants accordingly. Overlap weighting calculated between 0 and 1 determines the exact balance and optimized precision [23]. PS was applied so that migraine patients were weighted by the probability of PS and controls were weighted by the probability of 1-PS. We compared characteristics between migraine and control groups, as well as between two migraine subtypes and controls, before and after weighting.

A propensity score overlap weighted Cox proportional hazard regression model was used to estimate hazard ratios (HRs) and 95% confidence intervals (CIs) to investigate the association of migraine with CVD development. Crude (unadjusted) and overlap weighted models (controlled for age, sex, income, residential region, BMI, smoking status, drinking habit, SBP, DBP, fasting blood glucose, total cholesterol, hemoglobin, and CCI score) were calculated. The cumulative CVD incidence for each group was plotted using Kaplan‒Meier analysis and compared with the log-rank test.

Results that were associated with two-tailed P values of < 0.05 were considered statistically significant. All statistical analyses were conducted using SAS version 9.4 (SAS Institute Inc., Cary, NC, USA).

## Results

General characteristics of the patient population and control group were compared at baseline (Table 1). The proportion of all covariates between the migraine patients and controls was the same due to the use of covariate-matched cohorts (standardized difference = 0). Both migraine subgroups with and without aura also had the same covariate distribution as their respective control groups. A total of 3,360 (9.32%), 2,363 (6.55%), and 616 patients (1.71%) in the migraine group and 2,473 (6.86%), 21,790 (4.96%), and 582 patients (1.62%) in the control group developed stroke, ischemic heart disease, and heart failure, respectively.

**Table 1:** Baseline demographic and characteristics of study participants by presence of migraine

Characteristic		Migraine	Control	Standardized difference	Migraine with aura	Control	Standardized difference	Migraine without aura	Control	Standardized difference
Age (n, %)				0.00			0.00			0.00
40–44		1,375 (3.81)	1,375 (3.81)		155 (5.80)	155 (5.80)		1,220 (3.65)	1,220 (3.65)	
45–49		4,401 (12.20)	4,401 (12.20)		437 (16.38)	437 (16.38)		3,962 (11.87)	3,962 (11.87)	
50–54		5,921 (16.42)	5,921 (16.42)		515 (19.28)	515 (19.28)		5,405 (16.19)	5,405 (16.19)	
55–59		6,054 (16.79)	6,054 (16.79)		447 (16.75)	447 (16.75)		5,607 (16.80)	5,607 (16.80)	
60–64		5,503 (15.26)	5,503 (15.26)		363 (13.61)	363 (13.61)		5,140 (15.40)	5,140 (15.40)	
65–69		5,204 (14.43)	5,204 (14.43)		329 (12.32)	329 (12.32)		4,875 (14.60)	4,875 (14.60)	
70–74		4,056 (11.25)	4,056 (11.25)		247 (9.23)	247 (9.23)		3,809 (11.41)	3,809 (11.41)	
75–79		2,337 (6.48)	2,337 (6.48)		124 (4.64)	124 (4.64)		2,212 (6.63)	2,212 (6.63)	
80–84		947 (2.63)	947 (2.63)		42 (1.59)	42 (1.59)		904 (2.71)	904 (2.71)	
85+		260 (0.72)	260 (0.72)		11 (0.41)	11 (0.41)		249 (0.74)	249 (0.74)	
Sex (n, %)				0.00			0.00			0.00
Male		11,741 (32.56)	11,741 (32.56)		760 (28.47)	760 (28.47)		10,979 (32.89)	10,979 (32.89)	
Female		24,317 (67.44)	24,317 (67.44)		1,910 (71.53)	1,910 (71.53)		22,403 (67.11)	22,403 (67.11)	
Income (n, %)				0.00			0.00			0.00
1 (lowest)		6,632 (18.39)	6,632 (18.39)		474 (17.74)	474 (17.74)		6,158 (18.45)	6,158 (18.45)	
2		5,182 (14.37)	5,182 (14.37)		405 (15.16)	405 (15.16)		4,776 (14.31)	4,776 (14.31)	
3		5,859 (16.25)	5,859 (16.25)		422 (15.80)	422 (15.80)		5,437 (16.29)	5,437 (16.29)	
4		7,587 (21.04)	7,587 (21.04)		592 (22.15)	592 (22.15)		6,994 (20.95)	6,994 (20.95)	
5 (highest)		10,797 (29.94)	10,797 (29.94)		779 (29.15)	779 (29.15)		10,017 (30.01)	10,017 (30.01)	
Residential region (n, %)				0.00			0.00			0.00
Urban		14,123 (39.17)	14,123 (39.17)		1,041 (38.98)	1,041 (38.98)		13,080 (39.18)	13,080 (39.18)	
Rural		21,935 (60.83)	21,935 (60.83)		1,630 (61.02)	1,630 (61.02)		20,302 (60.82)	20,302 (60.82)	
Obesity^a^ (n, %)				0.00			0.00			0.00
Underweight		874 (2.42)	874 (2.42)		71 (2.64)	71 (2.64)		803 (2.40)	803 (2.40)	
Normal		13,057 (36.21)	13,057 (36.21)		1,002 (37.50)	1,002 (37.50)		12,054 (36.11)	12,054 (36.11)	
Overweight		9,724 (26.97)	9,724 (26.97)		737 (27.59)	737 (27.59)		8,986 (26.92)	8,986 (26.92)	
Obese I		11,298 (31.33)	11,298 (31.33)		783 (29.31)	783 (29.31)		10,513 (31.49)	10,513 (31.49)	
Obese II		1,105 (3.06)	1,105 (3.06)		79 (2.95)	79 (2.95)		1,026 (3.07)	1,026 (3.07)	
Smoking status (n, %)				0.00			0.00			0.00
Nonsmoker		29,181 (80.93)	29,181 (80.93)		2,227 (83.40)	2,227 (83.40)		26,949 (80.73)	26,949 (80.73)	
Past smoker		3,059 (8.48)	3,059 (8.48)		188 (7.05)	188 (7.05)		2,870 (8.60)	2,870 (8.60)	
Current smoker		3,818 (10.59)	3,818 (10.59)		255 (9.55)	255 (9.55)		3,562 (10.67)	3,562 (10.67)	
Drinking habit (n, %)				0.00			0.00			0.00
< 1 time a week		27,213 (75.47)	27,213 (75.47)		2,104 (78.80)	2,104 (78.80)		25,105 (75.21)	25,105 (75.21)	
≥ 1 time a week		8,844 (24.53)	8,844 (24.53)		566 (21.20)	566 (21.20)		8,277 (24.79)	8,277 (24.79)	
SBP (Mean, SD)		125.73 (14.85)	125.73 (7.61)	0.00	124.29 (14.80)	124.29 (7.57)	0.00	125.85 (14.84)	125.85 (7.62)	0.00
DBP (Mean, SD)		77.69 (9.54)	77.69 (4.85)	0.00	77.05 (9.68)	77.05 (4.88)	0.00	77.74 (9.53)	77.74 (4.84)	0.00
Fasting blood glucose (Mean, SD)		98.18 (24.43)	98.18 (11.21)	0.00	96.33 (22.41)	96.33 (10.14)	0.00	98.33 (24.57)	98.33 (11.30)	0.00
Total cholesterol (Mean, SD)		200.66 (34.15)	200.66 (17.06)	0.00	199.96 (34.16)	199.96 (16.74)	0.00	200.72 (34.15)	200.72 (17.09)	0.00
Hemoglobin (Mean, SD)		13.46 (1.27)	13.46 (0.65)	0.00	13.38 (1.24)	13.38 (0.65)	0.00	13.47 (1.28)	13.47 (0.65)	0.00
CCI score (Mean, SD)		0.69 (1.23)	0.69 (0.65)	0.00	0.65 (1.17)	0.65 (0.64)	0.00	0.69 (1.23)	0.69 (0.65)	0.00
Stroke (n, %)		3,360 (9.32)	2,473 (6.86)	0.09	280 (10.4)	172 (6.43)	0.15	3,079 (9.22)	2,301 (6.89)	0.09
Ischemic heart disease (n, %)		2,363 (6.55)	1,790 (4.96)	0.07	181 (6.77)	122 (4.57)	0.10	2,182 (6.54)	1,669 (5.00)	0.07
Heart failure (n, %)		616 (1.71)	582 (1.62)	0.01	36 (1.34)	37 (1.38)	0.00	580 (1.74)	545 (1.63)	0.01

^a^Obesity (body mass index, kg/m^2^) was categorized as < 18.5 (underweight), ≥ 18.5 to < 23 (normal), ≥ 23 to < 25 (overweight), ≥ 25 to < 30 (obese I), and ≥ 30 (obese II).

*CCI *Charlson Comorbidity Index, *DBP *Diastolic blood pressure, *SBP *Systolic blood pressure, *SD *Standard deviation

**Fig. 2:**
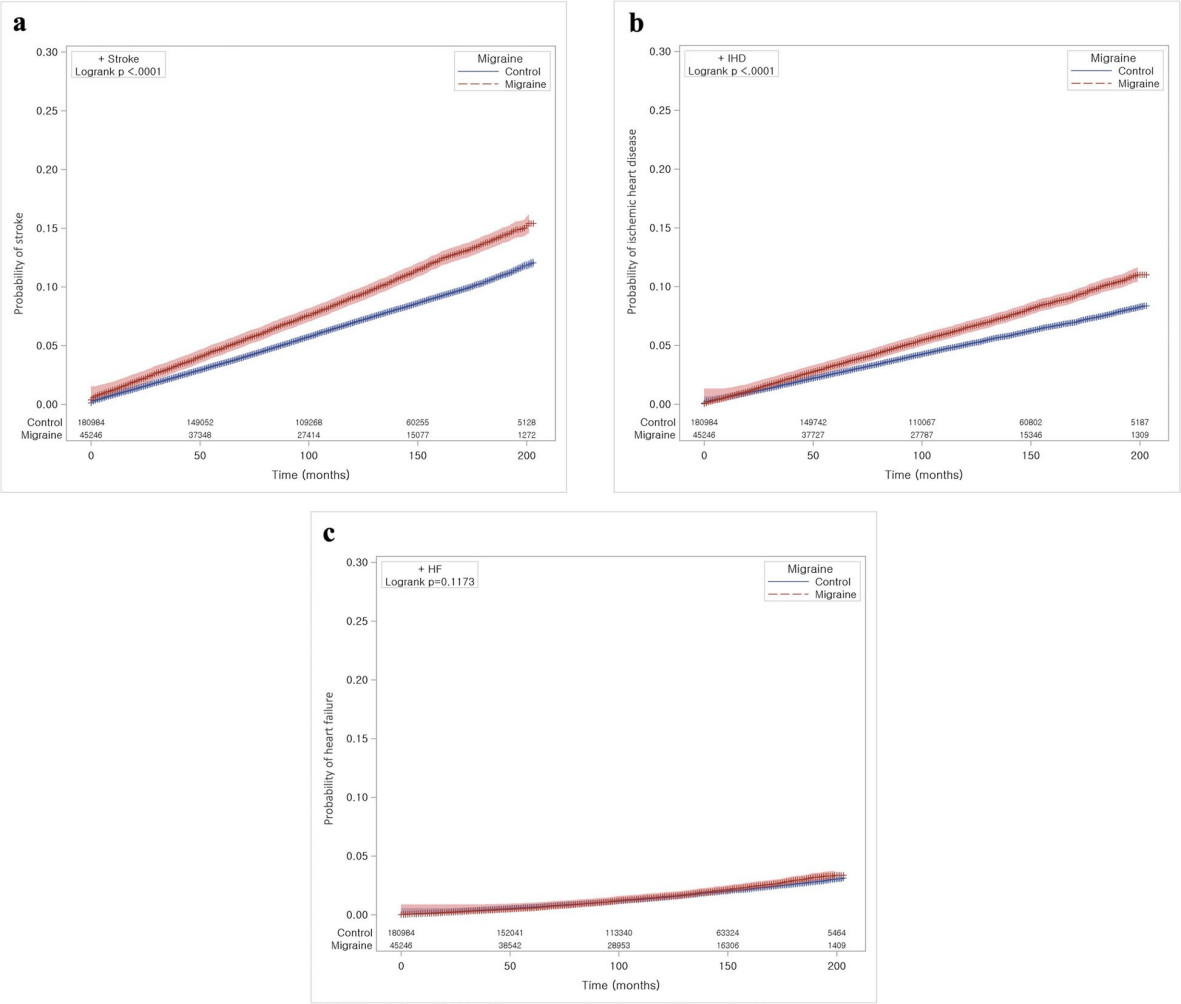
Kaplan‒Meier plots show the cumulative incidence of stroke (**a**), ischemic heart disease (**b**), and heart failure (**c**) in all groups of patients with migraine

The cumulative incidence of stroke, ischemic heart disease, and heart failure between the migraine and control groups was compared using Kaplan‒Meier survival curves and log-rank tests (Fig. 2). Patients with migraine developed more stroke and ischemic heart disease than those without migraine (log-rank P < 0.05). However, there were no significant associations with heart failure.

The incidence rates of stroke per 1000 person-years in the migraine group and control group were 10.25 and 7.64, respectively. After adjustment for covariates, the HR for stroke in patients with migraine compared with that in controls was 1.35 (95% CI = 1.31–1.39, Table 2). Patient groups who reported migraine with and without aura both showed higher stroke development than their controls (adjusted HR = 1.38, 95% CI = 1.29–1.48 and adjusted HR = 1.28, 95% CI = 1.24–1.32, respectively, Table 2).

**Table 2:** Incidence rates and hazard ratios for stroke among migraine and control groups

	No. of event / No. of total (%)	Follow-up duration (PY)	Incidence rate per 1000 (PY)	Incidence rate difference (95% CI)	Hazard ratios (95% CI)			
					Crude	P-value	Overlap weighted model^a^	*P*-value
Migraine and control group (*n* = 226,230)								
Migraine	4,216 / 45,246 (9.3)	411,173	10.25	2.61 (2.30 to 2.92)	1.34 (1.30–1.39)	< 0.001^*^	1.35 (1.31–1.39)	< 0.001^*^
Control	12,549 / 180,984 (6.9)	1,642,298	7.64		1		1	
Migraine with aura and control group (*n* = 16,815)								
Migraine with aura	352 / 3,363 (10.5)	33,193	10.60	3.89 (2.85 to 4.93)	1.58 (1.40–1.79)	< 0.001^*^	1.38 (1.29–1.48)	< 0.001^*^
Control	898 / 13,452 (6.7)	133,709	6.72		1		1	
Migraine without aura and control group (*n* = 209,414)								
Migraine without aura	3,864 / 41,883 (9.2)	377,967	10.22	2.50 (2.18 to 2.82)	1.32 (1.28–1.37)	< 0.001^*^	1.28 (1.24–1.32)	< 0.001^*^
Control	11,651 / 167,532 (7.0)	1,508,589	7.72		1		1	

^a^Adjusted for age, sex, income, residential region, obesity using body mass index, smoking status, drinking habit, systolic and diastolic blood pressure, fasting blood glucose, total cholesterol, hemoglobin, and Charlson Comorbidity Index scores

*CI *Confidence interval, *PY *Person-years

^*^Significance at P < 0.05

The incidence and HR of migraine for ischemic heart disease were significantly higher than those of controls (7.14 vs. 5.46 per 1000 person-years; adjusted HR = 1.31, 95% CI = 1.26–1.35, Table 3). Both migraine with aura and migraine without aura subtypes had significantly higher HRs for ischemic heart disease than the controls (adjusted HR = 1.21, 95% CI = 1.11–1.31 and adjusted HR = 1.27, 95% CI = 1.22–1.31, respectively, Table 3).

**Table 3:** Incidence rates and hazard ratios for ischemic heart disease among migraine and control groups

	No. of event / No. of total (%)	Follow-up duration (PY)	Incidence rate per 1000 (PY)	Incidence rate difference (95% CI)	Hazard ratios (95% CI)			
					Crude	P-value	Overlap weighted model^a^	*P*-value
Migraine and control group (*n* = 226,230)								
Migraine	2,973 / 45,246 (6.6)	416,160	7.14	1.69 (1.43 to 1.95)	1.31 (1.26–1.37)	< 0.001^*^	1.31 (1.26–1.35)	< 0.001^*^
Control	9,012 / 180,984 (5.0)	1,651,691	5.46		1		1	
Migraine with aura and control group (*n* = 16,815)								
Migraine with aura	227 / 3,363 (6.7)	33,886	6.70	2.05 (1.21 to 2.90)	1.44 (1.24–1.68)	< 0.001^*^	1.21 (1.11–1.31)	< 0.001^*^
Control	624 / 13,452 (4.6)	134,352	4.64		1		1	
Migraine without aura and control group (*n* = 209,414)								
Migraine without aura	2,746 / 41,883 (6.6)	382,261	7.18	1.66 (1.38 to 1.93)	1.30 (1.25–1.36)	< 0.001^*^	1.27 (1.22–1.31)	< 0.001^*^
Control	8,388 / 167,532 (5.0)	1,517,339	5.53		1		1	

*CI* confidence interval; *PY* person-years

^*^Significance at *P* < 0.05

^a^Adjusted for age, sex, income, residential region, obesity using body mass index, smoking status, drinking habit, systolic and diastolic blood pressure, fasting blood glucose, total cholesterol, hemoglobin, and Charlson Comorbidity Index scores

The incidence rates of heart failure in the migraine and control groups were 1.82 per 1000 person-years and 1.70 per 1000 person-years, respectively. Patients with migraine had an adjusted HR of 1.01 (95% CI = 0.95–1.08) for developing heart failure compared with controls (Table 4). There were also no significant differences in subsequent heart failure between the migraine and control groups when the migraine patients were divided into the migraine with and without aura subgroups (adjusted HR = 0.85, 95% CI = 0.7–1.02 and adjusted HR = 1.04, 95% CI = 0.97–1.10, respectively, Table 4).

**Table 4:** Incidence rates and hazard ratios for heart failure among migraine and control groups

	No. of event / No. of total (%)	Follow-up duration (PY)	Incidence rate per 1000 (PY)	Incidence rate difference (95% CI)	Hazard ratios (95% CI)			
					Crude	*P*-value	Overlap weighted model^a^	*P*-value
Migraine and control group (*n* = 226,230)								
Migraine	780 / 45,246 (1.7)	429,509	1.82	0.11 (-0.03 to 0.25)	1.07 (0.98–1.15)	0.117	1.01 (0.95–1.08)	0.668
Control	2,876 / 180,984 (1.6)	1,688,613	1.70		1		1	
Migraine with aura and control group (*n* = 16,815)								
Migraine with aura	46 / 3,363 (1.4)	35,005	1.31	-0.03 (-0.46 to 0.40)	0.98 (0.71–1.35)	0.896	0.85 (0.7–1.02)	0.084
Control	184 / 13,452 (1.4)	137,254	1.34		1		1	
Migraine without aura and control group (*n* = 209,414)								
Migraine without aura	2,746 / 41,883 (6.6)	382,261	7.18	0.13 (-0.02 to 0.27)	1.07 (0.99–1.16)	0.098	1.04 (0.97–1.10)	0.286
Control	8,388 / 167,532 (5.0)	1,517,339	5.53		1		1	

^a^Adjusted for age, sex, income, residential region, obesity using body mass index, smoking status, drinking habit, systolic and diastolic blood pressure, fasting blood glucose, total cholesterol, hemoglobin, and Charlson Comorbidity Index scores

*CI *Confidence interval, *PY *Person-years

^*^Significance at P < 0.05

 When analyzed in the subgroup stratified by age, sex, income, residential region, obesity, smoking status, alcohol consumption, blood pressure, fasting blood glucose, total cholesterol, hemoglobin, and CCI score, the results were similar to those observed in the main analysis (see Additional files 3, 4 and 5). In all subgroups, patients with migraine were more likely to develop stroke and ischemic heart disease than control participants, whereas there was no significant difference in the development of heart failure between the two groups.

## Discussion

The main finding of this study is that migraine is significantly associated with the development of subsequent stroke and ischemic heart disease, even after controlling for multiple cardiovascular risk factors. Specifically, patients with migraine had a 1.35-fold and 1.31-fold excess probability of subsequent stroke and ischemic heart disease than matched controls in the full adjustment model. However, we did not find a significant link between migraine and heart failure.

Consistent with our results, several studies exploring the relationship between migraine and CVDs have shown similar findings. A nationwide study in Taiwan consisting of 744 migraine patients and 617 matched comparison individuals showed that young migraineurs had a 2.5-fold higher risk of ischemic heart disease than nonmigraineurs (HR = 2.5, 95% CI = 1.78–3.52) [24]. A retrospective cohort study using data from US adults ≥66 years of age with Medicare health insurance showed that the adjusted HR among patients with versus without migraine was 1.20 (95% CI = 1.07–1.35) for ischemic stroke [25]. A similar observation was seen in middle-aged adults in the UK, in which migraine was associated with an increased risk for stroke and transient ischemic attack driven by a 2.2-fold and 2.4-fold increase in the risk of stroke and transient ischemic attack in 51,688 migraineurs and 51,688 matched controls [26].

In addition to population-based studies, our results are further supported by numerous meta-analyses. A previous meta-analysis of nine observational studies indicated that all types of migraine were associated with an increased risk of ischemic stroke (pooled relative risk [RR] = 1.73, 95% CI = 1.31–2.29), but it was only apparent (and twofold) among individuals who had migraine with aura (pooled RR = 2.16, 95% CI = 1.53–3.03). Additionally, migraine with aura (pooled RR = 2.08, 95% CI = 1.30–3.31), but not migraine without aura, seemed to be associated with a twofold increased risk of myocardial infarction [14]. Another meta-analysis of eleven prospective cohort studies involving 2,221,888 participants exhibited a significantly positive association, reporting that the pooled RR of ischemic stroke was 1.64 (95% CI = 1.22–2.20) for migraineurs compared with individuals who did not experience migraine [27]. In a recent meta-analysis of 16 cohort studies containing more than one million subjects, a history of migraine was related to a higher risk for stroke (adjusted HR = 1.41, 95% CI = 1.25–1.61) and myocardial infarction (adjusted HR = 1.41, 95% CI = 1.03–1.43) [28].

Since migraine usually affects young and middle-aged women, a considerable number of studies have mainly investigated migraine among women, particularly women younger than 45 years old. Those studies showed that migraine conferred an independent risk of cardiovascular outcomes, which were revealed to be stronger in women than in men. The aforementioned meta-analysis that provided a clear association of migraine with ischemic stroke also revealed that the RR was elevated to a greater extent among women (pooled RR = 2.08, 95% CI = 1.13–3.84) than among men (pooled RR = 1.37, 95% CI = 0.89–2.11) [14]. Other meta-analyses have also confirmed a double-increased risk of migraine for ischemic stroke in women but not in men [27, 29]. Moreover, a prospective study noted that the increased risk of stroke in women was most pronounced in younger patients aged 45 to 54 years [30]. Collectively, the majority of prior studies support a strong association between migraine and the risk of cardiovascular and cerebrovascular events that is more evident in women than in men. The authors of the prior literature on these robust associations between CVDs and migraines explained that because the prevalence of migraines is three times lower in men than in women, the association could be more uncertain for men. However, unexpectedly, in sex-stratified analyses in the current study, which were adjusted for the various traditional CVD risk factors, a sex difference in the associations between migraine and subsequent CVDs was not identified.

The association between migraine and CVD has been extensively explored but not completely clarified. Possible explanations for the increase in cardiovascular events by migraine are likely multifactorial. The first explanation may be attributed to the higher prevalence of several cardiovascular risk factors, such as smoking, hypertension, diabetes, and hyperlipidemia, in those with migraine [11, 28]. However, similar to some prior studies [13, 16], our adjusted analyses controlled for most of the conventional cardiovascular risk factors and thereafter showed a consistently significant association between migraine and stroke as well as ischemic heart disease. Second, numerous studies have noted patent foramen ovale (PFO)-mediated right-to-left shunting as a perpetrator for both migraine with aura and cryptogenic stroke [31, 32]. PFO occurs in 20–25% of adult people, particularly a maximum of 50% of migraineurs with aura. It may indicate a substrate for paradoxical emboli, eventually resulting in coronary and cerebral ischemic events [33]. While a meta-analysis of case control studies on the link between migraine and PFO supported an association of PFO with migraine with aura, no association was found in two population-based studies [34, 35]. The third mechanism linking migraine to CVD is related to the fact that those with migraine have a higher prevalence of a variety of hypercoagulable states, including endothelial dysfunction [36]. Hypercoagulability promotes both arterial and venous thrombosis, which may in turn cause cerebral infarction. Endothelial dysfunction, probably as a cause or consequence of migraine, is known to be mediated by oxidative stress. It was shown to be related to the early development of atherosclerosis but also to the activation of the coagulation pathway, enhanced inflammatory responses, and impaired vascular reactivity [37]. Furthermore, those with migraine were shown to have higher levels of platelet aggregation and von Willebrand factor [38–40]. These properties are more frequently observed in migraineurs and may explain the increased risk of cardiovascular and cerebrovascular events in individuals with migraine. Finally, it has been suggested that chronic use of nonsteroidal anti-inflammatory drugs (NSAIDs) in migraine patients may heighten CVD risk. Since NSAIDs are prevalently used during a migraine attack, migraineurs are thought to have greater exposure to NSAIDs than nonmigraineurs. Mounting evidence has provided information about cardiovascular risks with NSAIDs [41, 42]. However, these agents are widely used, and some are available in many countries without prescription. It is thus difficult to differentiate the potential adverse effects of NSAIDs from the biological effects of migraine itself through epidemiological studies. Therefore, the proposed mechanisms in this study warrant further investigation.

We acknowledge that our study has several limitations. First, there are concerns about the diagnostic accuracy of the database. The diagnosis of migraine, CVDs, and numerous covariates included in the analysis was completely defined by the ICD codes from the national database consisting of claim codes. Therefore, there is an inherent limitation that subjects who do not seek medical care for migraine may not be represented. In addition, migraine recorded using diagnostic codes in the claim data may be inaccurate. However, the validity of true diagnoses was increased by limiting the analyses to patients whose claims data included at least two corresponding diagnoses. Second, we were unable to evaluate information on a few recognized risk factors for CVD, such as lifestyle habits, physical activity or exercise, dietary risk factors, pregnancy, and hormonal contraceptive use, as well as risk factors for migraines such as comorbid psychiatric disorders. These unaccounted risk factors might modulate the association between migraine and CVD, and they need to be included in further research. Third, information was lacking regarding certain classes of medications used to control migraine, including triptans, ergotamines, and NSAIDs. Concerns have been raised regarding the cardiovascular safety of the use of migraine medications, especially triptans, owing to their vasoconstrictive ability [43]. In addition, the use of ergots has been reported to be related to white matter lesions [44]. Likewise, NSAIDs, which are commonly used by patients with migraine, are also related to an increased risk of cardiovascular events [41]. Accordingly, they might contribute to modifying the relationship between migraine and CVD; however, we did not consider these medications in our analysis. Fourth, further study is warranted after correcting the effect between dependent variables using multivariate analysis to confirm the contribution of migraine to CVD. Fifth, migraine usually affects young- to middle-aged women (25–55 years old); however, due to the nature of the database used in this study, only participants aged 40 or older were included in the analysis. Further research including participants in their 20 and 30 s is required to ascertain the contribution of migraine to CVD. Sixth, one potential source of bias when investigating the relationship between CVD and migraine is surveillance bias, which can go in different directions. Migraine patients, who frequently visit clinics, might have an increased chance of having a CVD identified. Last, since all subjects in this study were Korean, our data might not be generalizable to other ethnic groups.

Nevertheless, the current study has several strengths, including the use of multiple strategies to control for potential confounding and bias. Unlike the Danish study, our research relied on data from the Korean NIHS-health screening cohort. This cohort consisted of individuals who participated in health screening programs provided by the NHIS in Korea. The cohort database was constructed by selecting a sample from the 2002 to 2003 health screening participants, aged between 40 and 79 years in 2002, and following them up until 2013. The cohort consisted of 514,866 participants, representing a 10% random sample of all health screening participants during the specified years. Our study utilized this cohort, which included specific health issues and risk factors collected through questionnaires (such as smoking status, alcohol consumption, physical activity, medical and family history) and bioclinical laboratory results (including blood pressure, glucose level, lipid profile, hemoglobin level, urine analysis, creatinine level, liver enzymes, BMI, and waist circumference). Therefore, our cohort provided information on risk factors obtained through questionnaires and laboratory tests. As a results, we were able to adjust for well-known risk factors such as drinking habits, smoking status, blood pressure, BMI, fasting glucose level, and total cholesterol level, which were not accounted for in the previous study. We used a matched design to control for disparities in age, sex, income, and residential region. Additionally, we included many covariates in the statistical models to adjust for possible remaining confounding effects. The database used in this study includes information regarding well-known risk factors for CVDs, such as smoking status, alcohol intake, obesity status indicated by BMI, blood pressure, and the levels of fasting blood glucose and total cholesterol levels.

In conclusion, we demonstrated that patients with migraine harbored an approximately 30% higher probability of stroke and ischemic heart disease than nonmigraine patients, thereby implying that this group of patients represents a population that is susceptible to subsequent CVDs excluding heart failure. Our results warrant further validation, and the possible pathophysiology underlying these associations needs to be determined through further research.

## Supplementary Information

**Additional file 1.**

**Additional file 2.**

**Additional file 3.**

**Additional file 4.**

**Additional file 5.**

## Data Availability

The data that support the findings of this study are available from a Korean National Health Insurance Service-health screening cohort, but restrictions apply to the availability of these data, which were used under permission for the current study and so are not publicly available.

## Abbreviations

BMI: Body mass index
CCI: Charlson Comorbidity Index
CIs: Confidence intervals
CVD: Cardiovascular diseases
HRs: Hazard ratios
ICD: International Classification of Disease
NHIS: National Health Insurance Service
NSAIDs: Nonsteroidal anti-inflammatory drugs
PFO: Patent foramen ovale
PS: Propensity score
RR: Relative risk
