# Supplementary material for: A higher probability of subsequent stroke and ischemic heart disease in migraine patients: a longitudinal follow-up study in Korea
Source: J Headache Pain. 2023 Jul 31;24(1):98. doi: 10.1186/s10194-023-01632-y (PMC10391882; doi:10.1186/s10194-023-01632-y)
Supplement: Supplementary file 2 — Additional file 2. [file 10194_2023_1632_MOESM2_ESM.docx]

**Additional file 4** Subgroup analysis of incidence rates and hazard ratios for ischemic heart disease among migraine and control groups

| Subgroup | | No. of event /  No. of total (%) | | Follow-up duration (PY) | Incidence rate per 1000  (PY) | Incidence rate difference  (95% CI) | Hazard ratios (95% CI) | | | |
| --- | --- | --- | --- | --- | --- | --- | --- | --- | --- | --- |
|  |  |  |  |  |  |  | Crude | P-value | Overlap weighted model^†^ | P-value |
| Age <60 years old | | | | |  |  |  |  |  |  |
|  | Migraine | 1,103 / 22,260 (5.0) | | 239,038 | 4.61 | 1.59 (1.33 to 1.85) | 1.53 (1.42-1.64) | <0.001^*^ | 1.53 (1.44-1.62) | <0.001^*^ |
|  | Control | 2,899 / 89,040 (3.3) | | 957,976 | 3.03 |  | 1 |  | 1 |  |
| Age ≥60 years old | | | | |  |  |  |  |  |  |
|  | Migraine | 1,870 / 22,986 (8.1) | | 177,122 | 10.56 | 1.75 (1.25 to 2.25) | 1.20 (1.14-1.26) | <0.001^*^ | 1.20 (1.15-1.25) | <0.001^*^ |
|  | Control | 6,113 / 91,944 (6.6) | | 693,715 | 8.81 |  | 1 |  | 1 |  |
| Male | | | | |  |  |  |  |  |  |
|  | Migraine | 1,162 / 14,773 (7.9) | | 124,130 | 9.36 | 1.65 (1.09 to 2.21) | 1.21 (1.14-1.30) | <0.001^*^ | 1.20 (1.14-1.27) | <0.001^*^ |
|  | Control | 3,784 / 59,092 (6.4) | | 490,788 | 7.71 |  | 1 |  | 1 |  |
| Female | | | | |  |  |  |  |  |  |
|  | Migraine | 1,811 / 30,473 (5.9) | 292,030 | | 6.20 | 1.70 (1.42 to 1.98) | 1.38 (1.31-1.45) | <0.001^*^ | 1.38 (1.32-1.44) | <0.001^*^ |
|  | Control | 5,228 / 121,892 (4.3) | 1,160,903 | | 4.50 |  | 1 |  | 1 |  |
| Low income group | | | | |  |  |  |  |  |  |
|  | Migraine | 1,487 / 22,183 (6.7) | 205,660 | | 7.23 | 1.65 (1.27 to 2.02) | 1.30 (1.22-1.37) | <0.001* | 1.28 (1.22-1.34) | <0.001^*^ |
|  | Control | 4,541 / 88,732 (5.1) | 813,098 | | 5.58 |  | 1 |  | 1 |  |
| High income group | | | | |  |  |  |  |  |  |
|  | Migraine | 1,486 / 23,063 (6.4) | 210,500 | | 7.06 | 1.73 (1.37 to 2.09) | 1.32 (1.25-1.40) | <0.001* | 1.33 (1.27-1.40) | <0.001^*^ |
|  | Control | 4,471 / 92,252 (4.8) | 838,593 | | 5.33 |  | 1 |  | 1 |  |
| Urban residents | | | | |  |  |  |  |  |  |
|  | Migraine | 1,119 / 17,713 (6.3) | 162,271 | | 6.90 | 1.69 (1.28 to 2.09) | 1.32 (1.24-1.42) | <0.001^*^ | 1.34 (1.26-1.42) | <0.001^*^ |
|  | Control | 3,374 / 70,852 (4.8) | 647,776 | | 5.21 |  | 1 |  | 1 |  |
| Rural residents | | | | |  |  |  |  |  |  |
|  | Migraine | 1,854 / 27,533 (6.7) | 253,889 | | 7.30 | 1.69 (1.35 to 2.02) | 1.30 (1.23-1.37) | <0.001^*^ | 1.29 (1.23-1.34) | <0.001^*^ |
|  | Control | 5,638 / 110,132 (5.1) | 1,003,915 | | 5.62 |  | 1 |  | 1 |  |
| Underweight | | | | |  |  |  |  |  |  |
|  | Migraine | 65 / 1,083 (6.0) | 9,098 | | 7.14 | 1.30 (-0.49 to 3.09) | 1.23 (0.93-1.62) | 0.149 | 1.15 (0.92-1.44) | 0.229 |
|  | Control | 220 / 4,633 (4.7) | 37,642 | | 5.84 |  | 1 |  | 1 |  |
| Normal weight | | | | |  |  |  |  |  |  |
|  | Migraine | 931 / 16,367 (5.7) | 151,814 | | 6.13 | 1.72 (1.33 to 2.11) | 1.39 (1.29-1.50) | <0.001^*^ | 1.36 (1.28-1.45) | <0.001^*^ |
|  | Control | 2,652 / 65,781 (4.0) | 601,164 | | 4.41 |  | 1 |  | 1 |  |
| Overweight | | | | |  |  |  |  |  |  |
|  | Migraine | 774 / 12,230 (6.3) | 112,930 | | 6.85 | 1.63 (1.15 to 2.12) | 1.31 (1.21-1.42) | <0.001^*^ | 1.31 (1.22-1.40) | <0.001^*^ |
|  | Control | 2,321 / 48,297 (4.8) | 444,578 | | 5.22 |  | 1 |  | 1 |  |
| Obese | | | | |  |  |  |  |  |  |
|  | Migraine | 1,203 / 15,566 (7.7) | 142,318 | | 8.45 | 1.73 (1.24 to 2.22) | 1.26 (1.18-1.34) | <0.001^*^ | 1.27 (1.2-1.34) | <0.001^*^ |
|  | Control | 3,819 / 62,273 (6.1) | 568,307 | | 6.72 |  | 1 |  | 1 |  |
| Nonsmoker | | | | |  |  |  |  |  |  |
|  | Migraine | 2,293 / 36,689 (6.2) | 347,138 | | 6.61 | 1.63 (1.36 to 1.90) | 1.33 (1.27-1.39) | <0.001^*^ | 1.32 (1.27-1.37) | <0.001^*^ |
|  | Control | 6,749 / 144,679 (4.7) | 1,356,035 | | 4.98 |  | 1 |  | 1 |  |
| Past and current smoker | | | | |  |  |  |  |  |  |
|  | Migraine | 680 / 8,557 (7.9) | 69,022 | | 9.85 | 2.20 (1.45 to 2.94) | 1.29 (1.18-1.40) | <0.001^*^ | 1.26 (1.17-1.35) | <0.001^*^ |
|  | Control | 2,263 / 36,305 (6.2) | 295,656 | | 7.65 |  | 1 |  | 1 |  |
| Drinking habit <1 time a week | | | | |  |  |  |  |  |  |
|  | Migraine | 2,386 / 34,309 (7.0) | 337,042 | | 7.08 | 1.70 (1.41 to 1.99) | 1.32 (1.26-1.38) | <0.001^*^ | 1.31 (1.26-1.36) | <0.001^*^ |
|  | Control | 6,994 / 133,687 (5.2) | 1,300,025 | | 5.38 |  | 1 |  | 1 |  |
| Drinking habit ≥1 time a week | | | | |  |  |  |  |  |  |
|  | Migraine | 587 / 10,937 (5.4) | 79,118 | | 7.42 | 1.68 (1.08 to 2.28) | 1.29 (1.18-1.42) | <0.001^*^ | 1.30 (1.21-1.40) | <0.001^*^ |
|  | Control | 2,018 / 47,297 (4.3) | 351,666 | | 5.74 |  | 1 |  | 1 |  |
| SBP <140 mmHg and DBP <90 mmHg | | | | |  |  |  |  |  |  |
|  | Migraine | 1,978 / 34,167 (5.8) | 308,498 | | 6.41 | 1.73 (1.45 to 2.01) | 1.37 (1.30-1.44) | <0.001^*^ | 1.34 (1.28-1.40) | <0.001^*^ |
|  | Control | 5,612 / 133,186 (4.2) | 1,199,483 | | 4.68 |  | 1 |  | 1 |  |
| SBP ≥140 mmHg or DBP ≥90 mmHg | | | | |  |  |  |  |  |  |
|  | Migraine | 995 / 11,079 (9.0) | 107,662 | | 9.24 | 1.72 (1.13 to 2.31) | 1.23 (1.15-1.32) | <0.001^*^ | 1.25 (1.18-1.32) | <0.001^*^ |
|  | Control | 3,400 / 47,798 (7.1) | 452,208 | | 7.52 |  | 1 |  | 1 |  |
| Fasting blood glucose <100 mg/dL | | | | |  |  |  |  |  |  |
|  | Migraine | 1,838 / 30,432 (6.0) | 291,679 | | 6.30 | 1.68 (1.39 to 1.96) | 1.36 (1.29-1.44) | <0.001^*^ | 1.32 (1.26-1.38) | <0.001^*^ |
|  | Control | 5,170 / 116,995 (4.4) | 1,117,736 | | 4.63 |  | 1 |  | 1 |  |
| Fasting blood glucose ≥100 mg/dL | | | | |  |  |  |  |  |  |
|  | Migraine | 1,135 / 14,814 (7.7) | 124,481 | | 9.12 | 1.92 (1.39 to 2.46) | 1.27 (1.19-1.36) | <0.001^*^ | 1.29 (1.22-1.36) | <0.001^*^ |
|  | Control | 3,842 / 63,989 (6.0) | 533,955 | | 7.20 |  | 1 |  | 1 |  |
| Total cholesterol <240 mg/dL | | | | |  |  |  |  |  |  |
|  | Migraine | 1,425 / 23,277 (6.1) | 212,633 | | 6.70 | 1.79 (1.44 to 2.13) | 1.37 (1.29-1.45) | <0.001^*^ | 1.36 (1.3-1.44) | <0.001^*^ |
|  | Control | 4,176 / 93,778 (4.5) | 850,193 | | 4.91 |  | 1 |  | 1 |  |
| Total cholesterol ≥240 mg/dL | | | | |  |  |  |  |  |  |
|  | Migraine | 1,548 / 21,969 (7.0) | 203,527 | | 7.61 | 1.57 (1.18 to 1.96) | 1.26 (1.19-1.34) | <0.001^*^ | 1.25 (1.2-1.32) | <0.001^*^ |
|  | Control | 4,836 / 87,206 (5.5) | 801,498 | | 6.03 |  | 1 |  | 1 |  |
| Hemoglobin ≥12 for men and ≥10 for women (g/dL) | | | | | |  |  |  |  |  |
|  | Migraine | 2,929 / 44,527 (6.6) | 410,369 | | 7.14 | 1.69 (1.43 to 1.95) | 1.31 (1.26-1.37) | <0.001^*^ | 1.31 (1.26-1.35) | <0.001^*^ |
|  | Control | 8,850 / 177,670 (5.0) | 1,623,724 | | 5.45 |  | 1 |  | 1 |  |
| Hemoglobin <12 for men and <10 for women (g/dL) | | | | | |  |  |  |  |  |
|  | Migraine | 44 / 719 (6.1) | 5,791 | | 7.60 | 1.81 (-0.41 to 4.02) | 1.30 (0.93-1.81) | 0.128 | 1.15 (0.88-1.51) | 0.303 |
|  | Control | 162 / 3,314 (4.9) | 27,967 | | 5.79 |  | 1 |  | 1 |  |
| CCI scores = 0 | | | | |  |  |  |  |  |  |
|  | Migraine | 1,477 / 31,005 (4.8) | 288,553 | | 5.12 | 1.49 (1.23 to 1.74) | 1.41 (1.33-1.49) | <0.001^*^ | 1.39 (1.33-1.46) | <0.001^*^ |
|  | Control | 4,362 / 128,496 (3.4) | 1,200,983 | | 3.63 |  | 1 |  | 1 |  |
| CCI scores = 1 | | | | |  |  |  |  |  |  |
|  | Migraine | 636 / 6,264 (10.2) | 58,014 | | 10.96 | 1.13 (0.20 to 2.07) | 1.12 (1.02-1.22) | 0.016^*^ | 1.17 (1.08-1.26) | <0.001^*^ |
|  | Control | 1,833 / 20,572 (8.9) | 186,495 | | 9.83 |  | 1 |  | 1 |  |
| CCI scores ≥2 | | | | |  |  |  |  |  |  |
|  | Migraine | 860 / 7,977 (10.8) | 69,593 | | 12.36 | 1.70 (0.82 to 2.57) | 1.16 (1.08-1.25) | <0.001^*^ | 1.19 (1.11-1.26) | <0.001^*^ |
|  | Control | 2,817 / 31,916 (8.8) | 264,213 | | 10.66 |  | 1 |  | 1 |  |

CCI, Charlson Comorbidity Index; Confidence Interval; DBP, diastolic blood pressure; PY, person-years; SBP, systolic blood pressure.

^*^Significance at P <0.05.

Adjusted for age, sex, income, residential region, obesity using body mass index, smoking status, drinking habit, systolic and diastolic blood pressure, fasting blood glucose, total cholesterol, hemoglobin, and Charlson Comorbidity Index scores.
