# Supplementary material for: A higher probability of subsequent stroke and ischemic heart disease in migraine patients: a longitudinal follow-up study in Korea
Source: J Headache Pain. 2023 Jul 31;24(1):98. doi: 10.1186/s10194-023-01632-y (PMC10391882; doi:10.1186/s10194-023-01632-y)
Supplement: Supplementary file 4 — Additional file 4. [file 10194_2023_1632_MOESM4_ESM.docx]

**Additional file 1** Inclusion and exclusion criteria of the participants of two groups

|  | Inclusion criteria | Exclusion criteria |
| --- | --- | --- |
| Migraine group | - Individuals who were diagnosed with migraine without aura (ICD-10 code G430) and migraine with aura (ICD-10 code G431) at a minimum of 2 clinic visits between 2002 and 2019 | - Participants who were given a diagnosis of migraine (ICD-10 code G430 or G431) in 2002 - Participants who had no records of covariates - Participants who had a history of CVD before the index date |
| Control group | - Individuals who were not included in the initial migraine group during the period between 2002 and 2019 | - Participants who had been diagnosed with migraine once |

CVD, cardiovascular disease; ICD-10, International Classification of Disease, Tenth Revision.
