# Supplementary material for: A higher probability of subsequent stroke and ischemic heart disease in migraine patients: a longitudinal follow-up study in Korea
Source: J Headache Pain. 2023 Jul 31;24(1):98. doi: 10.1186/s10194-023-01632-y (PMC10391882; doi:10.1186/s10194-023-01632-y)
Supplement: Supplementary file 5 — Additional file 5. [file 10194_2023_1632_MOESM5_ESM.docx]

**Additional file 2** Definition or classification for the risk factors of cardiovascular disease used in this study

| Risk factor | Definition or classification |
| --- | --- |
| Income | The income groups were initially divided into 41 classes (one health aid class, 20 self-employment health insurance classes, and 20 employment health insurance classes. These groups were recategorized according to quintiles from class I (lowest income) to class 5 (highest income). Each quintile represents 20%. Finally, the income groups were divided into five classes (class 1 [lowest income]–5 [highest income]). |
| Residential region | The regions of residence were grouped into urban (Seoul, Busan, Daegu, Incheon, Gwangju, Daejeon, and Ulsan) and rural (Gyeonggi, Gangwon, Chungcheongbuk, Chungcheongnam, Jeollabuk, Jeollanam, Gyeongsangbuk, Gyeongsangnam, and Jeju) areas. |
| Body mass index | Body mass index (kg/m^2^) was categorized as <18.5 (underweight), ≥18.5 to <23 (normal), ≥23 to <25 (overweight), ≥25 to <30 (obese I), and ≥30 (obese II). |
| Smoking status | Current smoking status was defined as nonsmoker, past smoker, or current smoker according to the participant’s current smoking status |
| Drinking habit | Alcohol consumption was categorized as the frequency of alcohol consumption: <1 time a week, and ≥1 time a week |
| Systolic blood pressure | <120 mmHg, 120–139 mmHg, ≥140 mmHg |
| Diastolic blood pressure | <80 mmHg, 80–89 mmHg, ≥90 mmHg |
| Fasting blood glucose | <100 mg/dL, 100–125 mg/dL, ≥126 mg/dL |
| Total cholesterol | <200 mg/dL, 200-239 mg/dL, ≥240 |
| Hemoglobin | <13 g/dL or ≥13 g/dL in men  <12 g/dL or ≥13 g/dL in women |
| Charlson comorbidity index | 0, 1, 2, 3, ≥4 |
